# Family support, social security, commercial insurance, and aging anxiety among Chinese residents: a study based on the 2021 CGSS data

**DOI:** 10.3389/fpubh.2025.1577384

**Published:** 2025-04-02

**Authors:** He Gu, Qingli Tan, Yongxing Guo, Han He, Yu Zhang

**Affiliations:** ^1^School of Public Finance and Management, Yunnan University of Finance and Economics, Kunming, Yunnan, China; ^2^School of Tourism and Hospitality Management, Yunnan University of Finance and Economics, Kunming, Yunnan, China; ^3^School of Law and Political Science, Yunnan University of Finance and Economics, Kunming, Yunnan, China; ^4^Department of Nursing, Qujing Medical College, Qujing, Yunnan, China

**Keywords:** residents, aging anxiety, family support, social security, commercial insurance

## Abstract

**Objective:**

Aging anxiety has emerged as a significant issue affecting the mental health of Chinese residents and social stability. This study utilizes data from the 2021 Chinese General Social Survey (CGSS) to examine aging anxiety among Chinese residents and the effects of family support, social security, and commercial insurance on this anxiety.

**Methods:**

Descriptive statistics were first used to examine aging anxiety and its overall situation. Group differences in aging anxiety were analyzed using t-tests or ANOVA. A multiple linear regression assessed the influence of family support, social security, and commercial insurance on aging anxiety, controlling for socio-demographic variables. A robustness test further evaluated the stability of these effects by increasing control variables. Finally, heterogeneity analysis explored the differential impact of independent variables across groups by region, gender, education, and health status.

**Results:**

The overall aging anxiety level is moderate (3.39 ± 0.96), with health, psychological, and economic anxiety scores of 3.65 ± 1.12, 3.28 ± 1.14, and 3.24 ± 1.21, respectively. Higher anxiety was reported by rural residents, women, those with lower education, income, poor health, and no pension or medical insurance. Significant differences were observed across residence, gender, and health status, but not for work status, marital status, or number of children. Multiple linear regression revealed that family income and basic pension insurance significantly influence aging anxiety, while commercial insurance had no significant effect (*p* < 0.05).

**Conclusion:**

This study highlights moderate aging anxiety among Chinese residents, particularly in rural areas, women, low-income individuals, and those in poor health. To address this, policy should prioritize strengthening the social security system, expanding pension coverage, enhancing basic pension benefits, and improving living standards for rural and low-income urban residents. These actions are crucial for safeguarding psychological well-being and ensuring social stability.

## 1 Introduction

According to the United Nations’ World Social Report 2023 (1), the pace of global population aging is unprecedented. The proportion of individuals aged 65 and older is growing rapidly, particularly in developing countries. The global population of people aged 65 and above is expected to double over the next 30 years, increasing from its current level to 1.6 billion. By 2050, this demographic is projected to account for more than 16% of the world’s population, meaning one in every six people will be aged 65 or older. This demographic shift presents a series of challenges, particularly for the sustainability of social security and pension systems, especially in developing countries with limited economic resources. Aging also has profound implications for mental health, family structures, social relationships, and economic development.

As Chinese society enters the aging phase, shifts in the population structure have made aging an increasingly prominent social issue. In 2023, the number of people aged 60 and above in China reached 297 million, with an aging rate of 21.2%, marking the country’s entry into a moderately aging society. Between 2030 and 2035, the number of people aged 60 and above is expected to exceed 400 million, reaching 30%, thus transitioning to a heavily aging society. By 2050, the number of individuals aged 60 and older is projected to surpass 500 million, accounting for over 40% of the population, signifying the entry into a “super-aged” society (2). As China’s aging population continues to grow, the economic, health, and caregiving demands of the older adult are rising rapidly, while the distribution of social resources and the development of older adult care services remain relatively underdeveloped. This disparity has led to widespread concerns among Chinese residents regarding future pension security.

In this context, aging anxiety has emerged as a significant issue affecting the mental health of Chinese residents and social stability. Current research on aging anxiety and older adult mental health primarily originates from Western developed countries. However, due to differences in population dynamics and socio-economic development, the experiences of aging anxiety in non-Western developing countries differ significantly from those in Western nations. Despite the accelerating pace of aging in China, there is a notable gap in research on aging anxiety and related issues among residents of developing countries outside the Western context.

Aging anxiety refers to the anxiety or fear about phenomena related to the process of aging that may arise in the future (3). These concerns include worries about declines in health and physical functioning, financial security, cognitive ability, changes in physical appearance, and social losses. Aging anxiety, therefore, differs from other forms of anxiety, such as death anxiety, which is specifically focused on concerns about death, and general anxiety, which pertains more broadly to present-day issues rather than future, aging-specific concerns (4).

To assess aging anxiety, several measurement tools have been developed. Currently, two widely used instruments include the Anxiety about Aging Scale (AAS) and the Personal Anxiety Toward Aging Scale (PAAS). The AAS, developed by Lasher and Faulkender (3), is a multidimensional scale consisting of 20 items divided into four factors (five items per factor). Based on the content of the items within each factor, Lasher and Faulkender (3) identified four dimensions of aging anxiety: fear of aging, psychological concerns, appearance-related anxiety, and fear of loss. This scale has been translated into multiple languages and used in various countries to assess aging anxiety (5, 6). Some researchers have revised and adapted the AAS to create scales that are more tailored to specific national contexts of aging anxiety (7, 8).

Another conceptualization of aging anxiety is proposed by Kafer et al. (9), who developed the PAAS. This model views aging anxiety as a single underlying construct, assessed across seven indicators. These indicators are similar to those proposed by Lasher and Faulkender (3), such as concerns about changes in appearance, physical health, social relationship losses, and cognitive or decision-making autonomy. However, the PAAS also includes concerns about general future anxiety, economic dependency, and physical disabilities or mobility restrictions. Lynch (4) validated this single construct model of aging anxiety using the PAAS, supporting its use and arguing that a single construct model is simpler and more direct for assessing the construct validity of aging anxiety compared to the more complex multidimensional model.

Given the significant cultural differences in perceptions of aging, as well as the varying economic and social development conditions across countries, the content of aging anxiety may differ. Moreover, the use of multidimensional measurement scales increases the complexity of research. Therefore, it is essential that studies on aging anxiety measurement consider these contextual factors.

Biological, psychological, and social factors collectively determine an individual’s health status and emotional responses (10). Research on the factors influencing aging anxiety typically emphasizes various individual-level determinants, with particular attention paid to gender, age, and health. Lasher et al. (3) found that older adults exhibited higher levels of aging anxiety than younger adults. However, some studies have reported contrary findings (11, 12), while others have shown that the relationship between age and aging anxiety is not statistically significant (6). Kiely et al. (13) found that women generally experience higher levels of aging anxiety than men, likely due to women’s more disadvantaged positions in terms of work environment, social status, and economic conditions compared to men. Health is another significant factor influencing aging anxiety, with better health being associated with lower levels of anxiety (4, 27), although the strength of this association varies across different sources of anxiety.

From a social perspective, according to the Life Course Theory, an individual’s life trajectory is shaped by the interaction of social environments, historical contexts, policy systems, and personal experiences (28). Therefore, strengthening social support systems and optimizing social policies are effective means of reducing individual aging anxiety. Family support, social security, and commercial insurance are vital components of social support, social policies, and market mechanisms that help alleviate aging anxiety. In addition to personal security, family support serves as a key traditional support system. The strength of family support lies in its cohesion, providing direct responses to aging needs through intergenerational financial assistance, daily care, and emotional comfort, which can help mitigate aging anxiety to some extent. Sources of family support include marital status, family income, and the number of children. The greater the number of children, the more economic and emotional support the older adult receive, resulting in lower levels of aging anxiety (14). Social security and commercial insurance are also significant factors influencing aging anxiety in modern societies. Numerous studies have demonstrated that increases in income, social welfare, healthcare insurance, and improvements to social security systems can reduce individuals’ anxiety about survival and the future, including aging anxiety (15–20). In contrast to developed countries, many developing nations continue to primarily rely on family-based elder care, with social welfare levels often remaining low and social security systems underdeveloped. However, research on aging anxiety in developing countries, particularly concerning the relationship between family support, social security, and insurance, remains limited.

This study aims to analyze the aging anxiety of Chinese residents using data from the 2021 Chinese General Social Survey (CGSS) and to explore the relationship between aging anxiety and family support, social security, and commercial insurance. The study seeks to examine the psychological health challenges faced by China, a developing country, as it undergoes rapid aging, and to highlight the gap between social resources, psychological needs, and policy implementation.

## 2 Materials and methods

### 2.1 Data sources

This study utilizes data from the 2021 Chinese General Social Survey (CGSS), which is a representative, continuous cross-sectional social survey in China. It systematically and comprehensively collects data at multiple levels, including social, community, family, and individual levels. The 2021 CGSS data covers 19 provinces, municipalities, and autonomous regions in China, with a total of 8,148 samples. The core and thematic modules of the survey were administered to all respondents, while the additional health module from the East Asian Social Survey (EASS), the health module from the International Social Survey Program (ISSP), and the environmental module from the ISSP were randomly administered to one-third of the participants. The EASS health module includes questions on health status, healthcare services, social trust, and concerns about aging, among other topics. This study primarily uses data from the core module and the EASS health module. Samples with missing values for key variables were excluded, resulting in a final sample size of 2,028.

### 2.2 Variable selection and conceptual framework

#### 2.2.1 Dependent variable

The dependent variable in this study is aging anxiety. The measurement of aging anxiety is based on three questions derived from the health module of the 2021 Chinese General Social Survey (CGSS):

“I worry that I will not be able to take care of myself when I get old.”

“I worry that I will have to let others make decisions for me when I get old.”

“Being financially dependent on others is one of my biggest concerns about growing old.”

These questions correspond closely to the items related to physical disability/mobility, loss of cognitive ability or autonomy in decision-making, and financial dependence, as outlined in Kafer et al.’s (9) Aging Anxiety Scale. They represent three key dimensions of aging anxiety: health, mental well-being, and economic dependency. Given the absence of large-scale studies measuring aging anxiety in China, the use of the Aging Anxiety Scale (AAS) or the Personal Anxiety Toward Aging Scale (PAAS) would be overly complex and cumbersome. Furthermore, Lynch (4) demonstrated that a single construct measure based on the PAAS scale could effectively assess aging anxiety. Therefore, this study adopts the three aforementioned items to measure aging anxiety.

The responses to these three questions were collected using a Likert scale, with options ranging from “Strongly Agree,” “Agree,” “Neither Agree nor Disagree,” “Disagree,” to “Strongly Disagree.” These responses were reverse-coded, with values ranging from 5 to 1, where higher scores indicated stronger agreement (i.e., greater anxiety). The scores for the three items were summed and averaged, with higher average scores reflecting higher levels of aging anxiety. The final score ranged from 1 to 5, with higher values indicating greater anxiety.

The reliability and validity of the aging anxiety measure were tested. In terms of reliability, a Cronbach’s alpha coefficient above 0.7 is generally considered acceptable, and the reliability of the aging anxiety measure in this study was 0.761. Regarding validity, the Kaiser-Meyer-Olkin (KMO) value should exceed 0.5 for factor analysis to be deemed suitable. The KMO value for this measure was 0.677, indicating that factor analysis is appropriate for the data.

#### 2.2.2 Independent variables

Based on relevant research, the independent variables analyzed in this study are categorized into two main groups: family support variables and social security and commercial insurance variables.

Family support variables:

Marital status (0 = No spouse, 1 = Has spouse)

Number of children (0 = No children, 1 = 1–3 children, 2 = 4 or more children)

Annual household income [0 = Low income (≤ 50,000 RMB), 1 = Medium income (50,000–150,000 RMB), 2 = High income (≥ 150,000 RMB)]

Social security and commercial insurance variables:

Basic medical insurance (0 = Not enrolled, 1 = Enrolled)

Basic pension insurance (0 = Not enrolled, 1 = Enrolled)

Commercial medical insurance (0 = Not enrolled, 1 = Enrolled)

Commercial pension insurance (0 = Not enrolled, 1 = Enrolled)

#### 2.2.3 Control variables

The control variables in this study primarily include socio-demographic factors, such as residence, gender, age, education, employment, and health status.

Resident (0 = Rural, 1 = Urban)

Gender (0 = Male, 1 = Female)

Age [0 = Youth (18–44 years), 1 = Middle-aged (45–59 years), 2 = Older adult (60 years and above)]

Education (0 = Primary school or below, 1 = Middle school, 2 = High school/Technical school, 3 = University)

Employment status (0 = Non-agricultural employment, 1 = Farming, 2 = Currently unemployed)

Health status (0 = Unhealthy, 1 = Fair, 2 = Healthy).

Additionally, to conduct the robustness test, this study incorporates four additional control variables:

Number of family-owned houses (0 = No, 1 = one house, 2 = two or more houses).

Chronic health issues (0 = No, 1 = Yes)

Medical visit frequency in the past year (0 = Never, 1 = Occasionally, 2 = Frequently)

Social interaction frequency in the past year (0 = Never, 1 = Rarely, 2 = Occasionally, 3 = Frequently)

Physical Exercise Frequency in the past year (0 = Never, 1 = Occasionally, 2 = Frequently)

#### 2.2.4 Conceptual framework

In this study, we investigate the key factors influencing aging anxiety among Chinese residents. Based on the literature and relevant research, the factors influencing aging anxiety are categorized into three main types: family support variables, social security and commercial insurance variables, and socio-demographic control variables.

Family support variables include marital status, number of children, and annual household income, which primarily assess the level of familial support and economic security. Social security and commercial insurance variables include basic medical insurance, basic pension insurance, commercial medical insurance, and commercial pension insurance, aiming to understand the impact of both public and private insurance on aging anxiety. Lastly, control variables encompass resident, gender, age, education, employment, and health status, which help mitigate the influence of other potential factors affecting aging anxiety.

**Figure 1:**
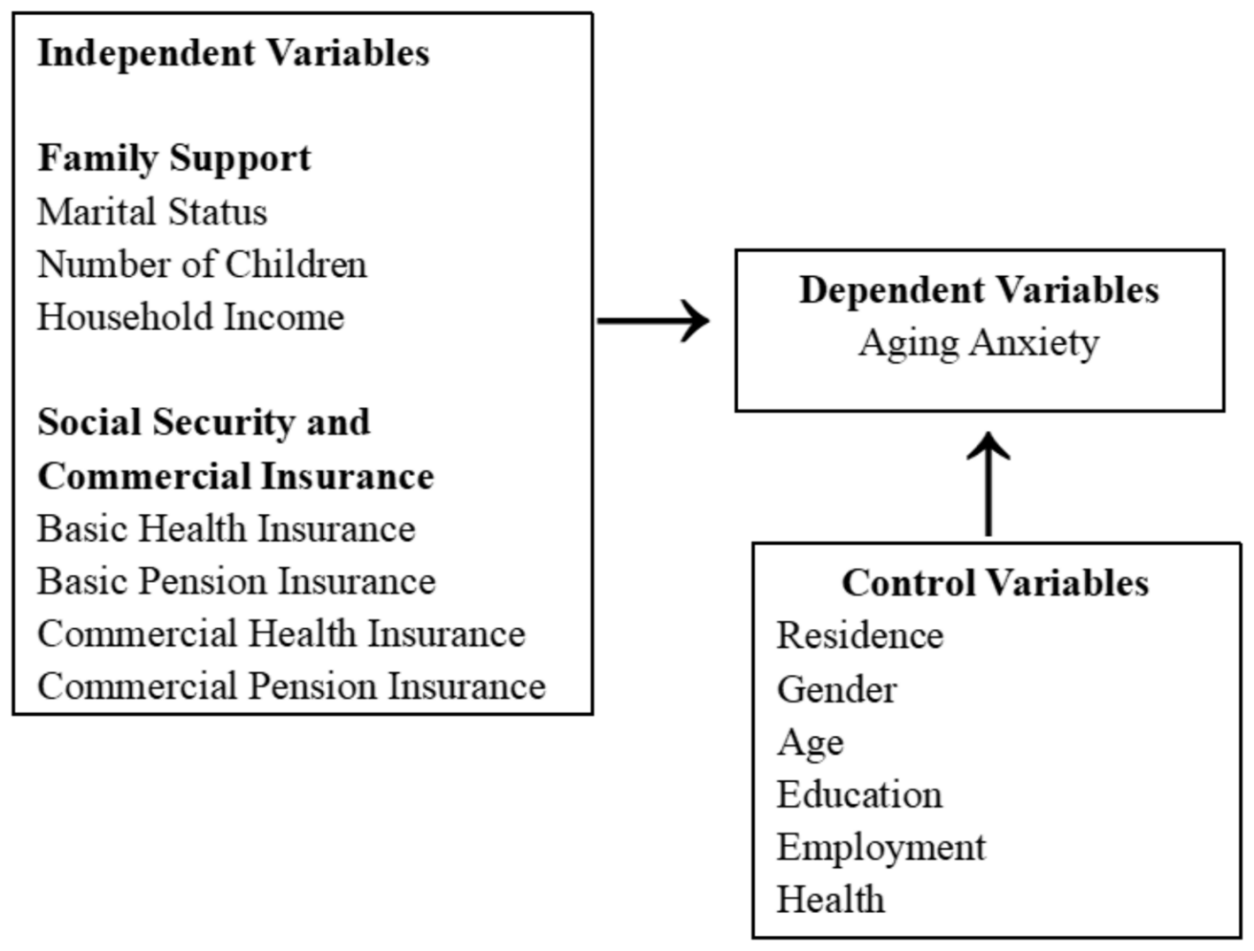
Conceptual framework.

Based on these variables, we will construct a conceptual framework to clearly illustrate the relationships and pathways between these factors (Figure 1).

### 2.3 Statistical analysis

Data analysis was conducted using SPSS 26.0, with a significance level set at *p* < 0.05. First, descriptive statistics were performed to examine the three items measuring aging anxiety and the overall situation. Next, the differences in aging anxiety across different groups were analyzed, with group comparisons conducted using t-tests or Analysis of Variance (ANOVA). Following this, a multiple linear regression analysis was performed to assess the influence of independent variables. The effect of family support, social security, and commercial insurance on aging anxiety among Chinese residents was analyzed while controlling for socio-demographic variables. Subsequently, a robustness test was conducted to further increase the number of control variables and assess the stability of the impact of the independent variables on aging anxiety. Finally, based on the previous analyses, heterogeneity analysis was carried out to examine the differential impact of independent variables on aging anxiety across groups defined by region, gender, education level, and health status.

## 3 Results

### 3.1 Demographic characteristics of the sample

A total of 2,028 participants were included in the study, comprising 956 males (47.1%) and 1,072 females (52.9%). Participants’ ages ranged from 18 years and older, with an mean age of 52.61 years. The distribution across age groups was generally balanced. In terms of education, 675 participants (33.3%) had completed primary school or lower, 591 (29.1%) had completed middle school, 368 (18.1%) had completed high school, and 394 (19.4%) had completed university. Regarding marital status, 245 participants (12.1%) were unmarried, while 1,783 (87.9%) were married.

### 3.2 Aging anxiety status of Chinese residents

Table 1 presents the aging anxiety status across three dimensions—health anxiety, mental anxiety, and economic anxiety—among the 2,028 Chinese residents surveyed. The mean scores for health anxiety, mental anxiety, and economic anxiety were 3.65, 3.28, and 3.24, respectively, all of which exceeded the neutral value of 3. This indicates that the respondents experienced a notable level of anxiety in terms of health, mental well-being, and economic concerns, with health anxiety being the most prominent. The overall aging anxiety score had a mean value of 3.39, suggesting that the overall level of aging anxiety among respondents was moderate to high.

**Table 1:** The scores of different dimensions of aging anxiety among 2,028 Chinese residents ($\bar{X}\pm S$).

Dimension	Sample size	Minimum value	Maximum value	$\bar{X}\pm S$
Health anxiety	2,028	1	5	3.65 ± 1.12
Psychological anxiety	2,028	1	5	3.28 ± 1.14
Economic anxiety	2,028	1	5	3.24 ± 1.21
Aging anxiety	2,028	1	5	3.39 ± 0.96

Table 2 presents the basic characteristics of the 2,028 Chinese residents surveyed, as well as the differences in aging anxiety levels across various characteristics. Aging anxiety scores were taken as the dependent variable, and univariate analysis was conducted to examine the relationship between aging anxiety and variables such as socio-demographic factors, family support, and social security and commercial insurance.

**Table 2:** Differences in aging anxiety levels by socio-demographic, family support, social security and commercial insurance characteristics.

Variable	Sample size	%	Aging anxiety score (Mean ± SD)	t/*F* value	*P* value
Resident				4.973b	0.000
Rural	1,202	59.27	3.48 ± 0.98		
Urban	826	40.73	3.26 ± 0.91		
Gender				-4.559b	0.000
Male	956	47.14	3.29 ± 0.98		
Female	1,072	52.86	3.48 ± 0.93		
Age				2.880c	0.056
Youth (18–44 years)	654	32.25	3.35 ± 0.87		
Middle-aged (45–59 years)	627	30.92	3.47 ± 0.95		
Older adult (60 years and above)	747	36.83	3.36 ± 1.04		
Education level				15.881c	0.000
Middle school or below	1,266	62.42	3.47 ± 0.98		
High school	368	18.15	3.28 ± 0.94		
University	394	19.43	3.20 ± 0.87		
Employment status				2.736c	0.065
Non-agricultural work	731	36.05	3.33 ± 0.90		
Agricultural work	368	18.15	3.45 ± 1.03		
Currently unemployed	929	45.81	3.41 ± 0.97		
Health status				28.892c	0.000
Unhealthy	379	18.69	3.64 ± 0.98		
Fair	596	29.39	3.48 ± 0.91		
Healthy	1,053	51.92	3.24 ± 0.95		
Marital status				−0.669b	0.503
No spouse	479	23.44	3.36 ± 0.93		
With spouse	1,549	76.56	3.40 ± 0.97		
Number of children				2.609c	0.074
No children	246	12.13	3.26 ± 0.84		
1–3 children	700	34.52	3.39 ± 0.95		
4 or more children	1,082	53.35	3.42 ± 1.00		
Household income				26.590c	0.000
Low (below 50,000 RMB)	1,021	50.35	3.52 ± 0.97		
Medium (50,000–150,000RMB)	737	36.34	3.33 ± 0.92		
High (above 150,000 RMB)	270	13.31	3.06 ± 0.93		
Basic medical insurance				0.172b	0.864
Not enrolled	108	5.33	3.40 ± 0.95		
Enrolled	1920	94.67	3.39 ± 0.96		
Basic pension insurance				2.495b	0.013
Not enrolled	483	23.82	3.48 ± 0.84		
Enrolled	1,545	76.18	3.36 ± 1.00		
Commercial medical insurance				2.391b	0.017
Not enrolled	1,745	86.05	3.41 ± 0.96		
Enrolled	283	13.95	3.26 ± 0.94		
Commercial pension insurance				2.527b	0.012
Not enrolled	1,873	92.36	3.40 ± 0.96		
Enrolled	155	7.64	3.20 ± 0.87		

b indicates t-value, c indicates F-value.

Univariate analysis of variables including resident, gender, education, health status, annual family income, participation in basic pension insurance, commercial medical insurance, and commercial pension insurance showed significant differences in aging anxiety levels (*p* < 0.05). Specifically, residents in rural areas exhibited the highest aging anxiety level, with a mean score of 3.48. Female residents reported higher aging anxiety levels (mean score: 3.48) compared to male residents. Residents with the lowest educational attainment (middle school or below) demonstrated the highest aging anxiety at 3.47, whereas those with the highest level of education (university degree) reported the lowest aging anxiety at 3.20. Furthermore, residents with lower family annual income had the highest aging anxiety level (3.52). Additionally, residents in poor health had the highest aging anxiety score (3.64), the highest among all groups. Residents not enrolled in basic pension insurance, commercial medical insurance, or commercial pension insurance had the higher aging anxiety scores, with mean values of 3.48, 3.41, and 3.40, respectively. Although aging anxiety levels across different age groups showed a near-significant difference (*p* < 0.05), when age was treated as a continuous variable, the Pearson correlation coefficient between aging anxiety and age was found to be −0.011, with a *p*-value of 0.626. This suggests that there is no significant linear relationship between age and aging anxiety, indicating that age does not exert a significant impact on aging anxiety levels. Other variables, such as work status, marital status, number of children, and participation in basic medical insurance, did not show statistically significant differences in aging anxiety levels (*p* > 0.05).

### 3.3 Factors influencing aging anxiety among Chinese residents

In Table 3, based on the results of the univariate analysis of aging anxiety among Chinese residents, variables with statistically significant differences, including household annual income, basic pension insurance, commercial medical insurance, commercial pension insurance, as well as region, gender, education level, and health status, were incorporated into a multiple linear regression model for further analysis.

Model 1 included household income, basic pension insurance, commercial medical insurance, and commercial pension insurance, focusing primarily on the influence of household annual income, basic pension insurance, and commercial insurance on aging anxiety. Model 2 added sociodemographic variables such as region, gender, education level, and health status as control variables, further analyzing the impact of family support, social security, and commercial insurance on aging anxiety. To conduct the robustness test, Model 3 further increased the number of control variables, including five additional variables, to assess the stability of the effects of family support, social security, and commercial insurance on aging anxiety. The omnibus test results for Models 1, 2, and 3 were all highly significant (*p* < 0.001), indicating that all three models have strong explanatory power.

Results from Model 1 indicated that household annual income within family support had a highly significant impact on aging anxiety among Chinese residents (*p* < 0.001). Household income was negatively correlated with aging anxiety, with every one-unit increase in household annual income resulting in a 0.208-unit decrease in aging anxiety levels, suggesting that higher household income is associated with lower aging anxiety. Among the three insurance variables (basic pension insurance, commercial medical insurance, and commercial pension insurance), basic pension insurance had a significant effect on aging anxiety (*p* < 0.05), with participation in basic pension insurance reducing aging anxiety by 0.109 units. However, both commercial medical insurance and commercial pension insurance had no significant effects on aging anxiety (*p* > 0.05).

Results of Model 2 showed that, after controlling for sociodemographic variables, the effect of household income remained highly significant (*p* < 0.001), while the impact of basic pension insurance was also significant (*p* < 0.05), and the effects of commercial medical insurance and commercial pension insurance were still not significant (*p* > 0.05).

In Model 3, with an increased number of control variables, the regression coefficients for household income and pension insurance showed slight changes, but the effect of household income remained highly significant (*p* < 0.001), while the impact of basic pension insurance remained significant (*p* < 0.05), and the effects of commercial medical insurance and commercial pension insurance remained insignificant (*p* > 0.05). This indicates that the regression coefficients and significance of household income and basic pension insurance largely remained consistent even after controlling for more variables, suggesting that the results are robust (Table 3).

**Table 3:** The multiple linear regression analysis of factors influencing aging anxiety among Chinese residents.

Influence variable	Model 1			Model 2			Model 3		
	*β*	Sx	*P-*value	*β*	Sx	*P-*value	*β*	Sx	*P-*value
Constant	4.029	0.126	0.000	4.177	0.155	0.000	4.106	0.232	0.000
Household income	−0.208	0.031	0.000	−0.131	0.034	0.000	−0.112	0.035	0.001
Basic pension insurance	−0.109	0.049	0.027	−0.109	0.049	0.027	−0.103	0.049	0.037
Commercial medical insurance	0.010	0.076	0.898	0.067	0.076	0.374	0.087	0.076	0.253
Commercial pension insurance	−0.111	0.098	0.254	−0.132	0.096	0.172	−0.141	0.096	0.144
Control variables									
Resident				−0.099	0.048	0.037	−0.106	0.049	0.029
Gender				0.168	0.042	0.000	0.160	0.042	0.000
Education level				−0.026	0.032	0.412	−0.018	0.033	0.583
Health status				−0.169	0.028	0.000	−0.155	0.032	0.000
Number of family-owned houses							−0.120	0.046	0.008
Chronic health issues							0.021	0.051	0.688
Medical visit frequency							0.100	0.036	0.005
Social interaction frequency							0.012	0.019	0.511
Physical exercise frequency							0.037	0.026	0.151

Dependent variable: aging anxiety. β refers to the standardized regression coefficient, Sx refers to the standard error of the coefficient, and *P*-value refers to the significance level.

### 3.4 Heterogeneity analysis

The results of the previous univariate analysis reveal significant differences in aging anxiety levels across various social groups among Chinese residents. Furthermore, the findings from the multiple linear regression analysis indicate that both household income and basic pension insurance are significant factors in reducing aging anxiety among Chinese residents. Previous research suggests that individual characteristics may influence the extent of aging anxiety. Consequently, the question arises: do household income and basic pension insurance exert significantly different effects on aging anxiety across different demographic groups? To address this, the following section presents a heterogeneity analysis.

#### 3.4.1 Resident heterogeneity

As shown in Table 4, household income plays a significant role in alleviating aging anxiety among both rural and urban residents, with its effect being stronger in urban areas, as indicated by the larger absolute value of the coefficient. This suggests that income growth has a more pronounced impact on mitigating aging anxiety in urban residents. In contrast, basic pension insurance significantly reduces aging anxiety in rural areas but does not have a significant effect in urban areas. This discrepancy may be due to the fact that rural residents are more reliant on basic pension insurance, whereas urban residents are more likely to depend on other forms of retirement security, such as commercial insurance or personal savings.

**Table 4:** Heterogeneity analysis of the impact of household income and basic pension insurance on aging anxiety by resident and gender among Chinese residents.

	Aging anxiety			
Variable	Resident		Gender	
	Rural	Urban	Male	Female
Household income	−0.124 (−2.635)^***^	−0.172 (−3.489)^***^	−0.157^***^ (−3.065)	−0.122^***^ (−2.672)
Basic pension insurance	−0.171 (−2.767)^***^	0.005 (0.066)	−0.164^**^ (−2.199)	−0.057 (−0.874)
Control variables	Controlled		Controlled	
R^2^	0.052	0.047	0.045	0.056
Sample size	1,202	826	956	1,072

*, **, *** indicate significance at the 10%, 5%, and 1% levels, respectively. The values in parentheses are t-values.

#### 3.4.2 Gender heterogeneity

Table 4 indicates that the effect of household income on alleviating aging anxiety is more pronounced among male residents, as evidenced by the larger absolute value of the coefficient. This suggests that income increases have a more significant impact on reducing aging anxiety among men. Additionally, basic pension insurance significantly reduces aging anxiety among men but does not have a significant effect on women. This may be attributed to the fact that men often bear greater financial responsibilities within the family, making them more reliant on pension insurance.

#### 3.4.3 Educational attainment heterogeneity

As presented in Table 5, the effect of household income on reducing aging anxiety varies significantly across different educational attainment groups. Household income has a significant effect on alleviating aging anxiety among residents with lower education levels (middle school or below) and a highly significant effect among those with higher education levels (university). However, its effect is not significant for residents with moderate education levels (high school). Meanwhile, basic pension insurance significantly reduces aging anxiety among residents with lower education levels but has no significant effect on those with moderate or higher educational attainment.

**Table 5:** Heterogeneity analysis of the impact of household income and basic pension insurance on aging anxiety by education and health among Chinese residents.

	Aging anxiety					
Variable	Education level			Health status		
	Middle school or below	High school	University	Unhealthy	Fair	Healthy
Household income	−0.100^**^ (−2.162)	−0.096 (−1.277)	−0.256^***^ (−3.892)	−0.127 (−1.320)	−0.128^**^ (−2.110)	−0.150^***^ (−3.313)
Basic pension insurance	−0.137^**^ (−2.130)	−0.168 (−1.408)	0.038 (0.374)	0.038 (0.315)	−0.137 (−1.537)	−0.117 (0.87)
Control variables	Controlled			Controlled		
R^2^	0.051	0.025	0.060	0.037	0.057	0.025
Sample size	1,266	368	394	379	596	1,053

*, **, *** indicate significance at the 10%, 5%, and 1% levels, respectively. The values in parentheses are t-values.

#### 3.4.4 Health condition heterogeneity

According to Table 5, household income significantly reduces aging anxiety among residents with moderate health conditions and has a highly significant effect on those in good health. However, its effect is not significant for residents with poor health. In contrast, basic pension insurance does not have a significant effect on aging anxiety among residents in any health condition group.

In summary, household income significantly alleviates aging anxiety across different regions and genders, with a stronger effect observed among residents with either lower or higher educational attainment, as well as those in moderate or good health. Meanwhile, basic pension insurance is more effective in reducing aging anxiety among rural residents, men, and those with lower educational attainment.

## 4 Discussion

This study found that Chinese residents currently experience moderate to high levels of aging anxiety, with concerns related to health, mental well-being, and economic factors all exceeding the average score. Health anxiety, in particular, emerged as the most prominent. These findings are consistent with those of Nie et al. (21), who conducted a survey of 1,126 rural residents across 11 provinces in China between 2019 and 2021.

From a socio-demographic perspective, the study identified that aging anxiety is more pronounced among rural residents, women, individuals with lower education levels, and those in poorer health. These results align with research conducted in other countries, which suggests that groups experiencing higher levels of aging anxiety share similar characteristics across different countries or regions (6, 22, 23). Notably, women exhibit more significant aging anxiety, a pattern observed in both Western and non-Western contexts (6, 24). This is likely due to women’s generally lower economic and social status, as well as the heavier caregiving responsibilities they bear, which contribute to elevated levels of anxiety regarding health, mental well-being, and economic concerns related to aging (25, 29).

Personal health status also plays a significant role in aging anxiety among Chinese residents. Specifically, for every one-unit increase in health status, aging anxiety decreases by 0.169 units, highlighting the strong relationship between health and aging anxiety. This finding is consistent with research from other countries, where the decline in physical health and associated health issues are among the most significant factors contributing to aging anxiety (4).

Additionally, the study found that resident significantly influences aging anxiety, with rural residents in China experiencing markedly higher levels of aging anxiety compared to their urban counterparts. This disparity reflects the characteristics of China as a developing country, where there has historically been a significant gap in living standards and social security benefits between urban and rural populations. For instance, in 2024, the per capita disposable income for urban residents in China was 54,188 yuan, whereas rural residents’ per capita disposable income was only 23,119 yuan—less than half of that of urban residents (30). Moreover, there is a substantial urban–rural divide in China’s pension system, a result of the long-standing urban–rural dual structure and the segregated design of the pension insurance system. At present, China operates two types of basic pension insurance: urban employee pension insurance and urban–rural resident pension insurance. While most urban residents participate in the former, a portion of urban residents and nearly all rural residents participate in the latter. However, the benefits provided by these two programs differ significantly. For example, in 2023, the average monthly pension for urban employee pension insurance was around 3,000 yuan, while the average monthly pension for urban–rural resident pension insurance was only approximately 200 yuan—less than 1/10 of the former (31). Consequently, rural residents tend to have lower social welfare and social security levels and face greater risks in old age, which may contribute to their heightened aging anxiety.

Furthermore, residents with lower education levels in China also report higher levels of aging anxiety, a finding that is consistent with studies conducted in other countries (5). However, the effect of education level on aging anxiety was found to be insignificant in this study, suggesting that education may serve more as an intermediary variable rather than a direct factor.

Interestingly, the study also shows that age does not significantly affect aging anxiety among Chinese residents. In fact, the middle-aged group exhibited higher levels of aging anxiety compared to other age groups, a finding that mirrors the results of Kruger (32). However, the relationship between age and aging anxiety is complex and likely influenced not only by socioeconomic factors but also by cultural considerations.

In general, family support, social security, and commercial insurance play crucial roles in alleviating aging anxiety, with family support being particularly prominent in developing countries where the pension system remains underdeveloped (33, 34). In China, as a developing nation, residents have long relied on family support, particularly from children, to address pension needs. This reliance is rooted in both traditional Chinese cultural values and the relatively underdeveloped pension system. However, in this study, the influence of family support, especially from spouses and children, appears to be minimal, with a more substantial effect stemming from economic support within the family. Household annual income demonstrates a significant negative impact on aging anxiety, with this effect remaining stable across different models, indicating that increasing household income can markedly reduce aging anxiety. Specifically, for every one-unit increase in household income, aging anxiety decreases by 0.215 units. This highlights the primary role of family support in alleviating aging anxiety through economic assistance, while its influence in other areas remains less pronounced. These findings are consistent with the research by Hao et al. (35), which examined aging concerns among the parents of only children in China. Their study revealed that the effect of child support on aging anxiety is predominantly economic, with little impact from the number of children or spousal support. This suggests a weakening of traditional family support structures. Overall, with China’s ongoing social and economic development, the role of family in older adult support is evolving, with its impact on aging anxiety increasingly reflecting material aspects rather than emotional or caregiving support.

In contemporary society, social security and commercial insurance are essential mechanisms for addressing aging anxiety, particularly regarding older adult health concerns (36). In this study, among the four components of social security and commercial insurance, participation in basic pension insurance is found to significantly reduce aging anxiety, even after controlling for household income and demographic variables. Residents who participate in basic pension insurance report significantly lower levels of aging anxiety compared to those who do not, with participation resulting in a reduction of aging anxiety by 0.117 units. This finding aligns with research by Xu et al. (37) on rural residents in China. In contrast, participation in basic medical insurance does not significantly affect aging anxiety, suggesting that basic medical insurance does not have a substantial impact on aging anxiety among Chinese residents. This discrepancy may be attributed to the different functional orientations and systemic designs of these two social insurance programs.

From a functional perspective, basic pension insurance is primarily designed to ensure income security for the older adult, thereby maintaining a certain standard of living after retirement. It is directly tied to the economic situation and quality of life in old age. As a result, when individuals lack confidence in their future economic security, their levels of aging anxiety tend to increase. The absence of participation in basic pension insurance may lead to concerns about insufficient future income, thereby heightening anxiety. In contrast, basic medical insurance primarily addresses medical expenses and current health needs. While it alleviates the financial burden of healthcare, it does not fundamentally resolve concerns about income. Even in the absence of basic medical insurance, healthcare issues are often short-term, and many individuals can still access necessary services through alternative means, such as commercial medical insurance or support from family and friends. As such, the anxiety associated with not having basic medical insurance is less pronounced compared to the absence of pension insurance.

From a systemic design perspective, China’s basic pension insurance system requires long-term contributions (at least 15 years), involves a complex benefit calculation, and is influenced by macroeconomic factors such as population aging and pension deficits. This complexity leads to a lower predictability of future benefits, which may generate anxiety among participants regarding whether they will receive the full benefits they have contributed to. Additionally, the benefits of China’s basic resident pension insurance are heavily dependent on fiscal subsidies, which are closely linked to local economic conditions, thereby increasing uncertainty. In contrast, China’s basic medical insurance system is more immediate and relatively certain, with clearly defined reimbursement rules that address current health risks. Participants are generally more concerned with the present reimbursement process than with potential future policy changes. Whether or not individuals are anxious about their pensions, medical treatment is universally recognized as a basic necessity. Enrollment in medical insurance is often regarded as a “passive necessity” rather than an active choice, which likely explains its weaker association with aging anxiety.

Moreover, public awareness of the two types of insurance, social psychology surrounding these programs, and other factors may also contribute to the differing impacts of basic pension insurance and basic medical insurance on aging anxiety. Notably, as discussed earlier, the study found that health-related anxiety among Chinese residents is the most prominent aspect of aging anxiety. Additionally, personal health status exerts a highly significant influence on aging anxiety in China. In modern society, health insurance plays a pivotal role in addressing various health issues for the general population (38). Although this study shows that basic medical insurance does not significantly affect aging anxiety in China, the impact of basic medical insurance on aging anxiety and related psychological health concerns warrants further investigation.

Regarding commercial insurance, the study observed significant differences in aging anxiety between residents who participate in commercial health insurance and commercial pension insurance, with those enrolled in these programs reporting lower levels of aging anxiety compared to those who are not. However, regression analysis revealed that the impact of participation in commercial health insurance and commercial pension insurance on aging anxiety was not statistically significant. This could be attributed to the moderating effect of household income on the influence of commercial insurance, as well as the overlap between basic pension insurance and commercial health or pension insurance. In China, individuals who purchase commercial insurance often also participate in basic social insurance, which may attenuate the effects of commercial insurance. Moreover, the development of commercial insurance in China is still in its early stages, with numerous issues related to public awareness and trust in these programs. Even if individuals are enrolled in commercial insurance, they may still harbor concerns about the future delivery of benefits, thus limiting its potential to effectively reduce aging anxiety (26).

It is widely acknowledged that social security and commercial insurance programs play a critical role in alleviating psychological health issues, including aging anxiety (39, 40). However, the effectiveness of these programs is contingent upon the rationality of their compensation mechanisms (41). Therefore, the specific design and implementation of these programs can significantly influence their impact on aging anxiety.

## 5 Conclusion

This study reveals that Chinese residents experience a moderately high level of aging anxiety, underscoring the need to address this issue within the context of China’s aging population. Particular attention should be directed toward residents in rural areas, women, individuals with low family income, and those in poor health.

With the ongoing changes in China’s socio-economic structure, the vulnerabilities of traditional forms of older adult support have become increasingly evident. The role of family-based support has weakened to some extent, with the influence of spouses and children becoming less significant. Instead, family support now primarily manifests in economic terms. While the traditional family support system should continue to play its role, there is a need to further strengthen institutional supports, especially in the area of pension insurance.

From a policy perspective, enhancing household income, strengthening health management, and promoting the rational development of the social security system are effective strategies for mitigating aging anxiety. For developing countries like China, where population aging and economic pressures are intensifying, it is essential to not only utilize traditional forms of older adult support but also adopt comprehensive measures to promote social equity and harmony. These measures should include optimizing the design of social security programs and the equitable distribution of pension resources. The policy focus should prioritize increasing investments in the social security system, expanding pension coverage, increasing the benefits of basic pension insurance, and specifically improving the living standards of rural and low-income urban residents. Such actions are essential for safeguarding the psychological well-being of the population and ensuring long-term social stability.

## Data Availability

The original contributions presented in the study are publicly available. This data can be found at: http://www.cnsda.org/index.php?r=projects/view&id=65635422.
